# Supplementary material for: Dietary Intake of Flavonoids Associated with Sleep Problems: An Analysis of Data from the National Health and Nutrition Examination Survey, 2007–2010
Source: Brain Sci. 2023 May 29;13(6):873. doi: 10.3390/brainsci13060873 (PMC10296398; doi:10.3390/brainsci13060873)
Supplement: Supplementary file 1 [file brainsci-13-00873-s001.zip › brainsci-2327150-supplementary.pdf]

Supplementary Material

Table S1. The differences in the sleep-related questionnaires between NHANES 2007-2010 cycle and 2017-2018 cycle

	2007-2010 cycle	2017-2018 cycle
**Sleep duration**	SLD010H - How much sleep do you get (hours)?	SLD012 - Number of hours usually sleep on weekdays or workdays?<br>SLD013 - Number of hours usually sleep on weekends or non-workdays?
**Sleep disorders**	SLQ050 - Ever told doctor had trouble sleeping?<br>SLQ060 - Ever told by doctor have sleep disorder?	SLQ050 - Ever told doctor had trouble sleeping?

SLD010H, SLD012, SLD013, SLQ050, SLQ060 means the codes of the structure questions from NHANES.
